# Cellular Attachment and Entry Factors for Chikungunya Virus

**DOI:** 10.3390/v11111078

**Published:** 2019-11-19

**Authors:** Barbara S. Schnierle

**Affiliations:** Department of Virology, Paul-Ehrlich-Institut, 63225 Langen, Germany; Barbara.schnierle@pei.de

**Keywords:** alphavirus, chikungunya virus, receptor

## Abstract

Chikungunya virus (CHIKV) is clinically the most relevant member of the *Alphavirus* genus. Like alphaviruses in general, CHIKV has the capacity to infect a large variety of cells, tissues, and species. This broad host tropism of CHIKV indicates that the virus uses a ubiquitously expressed receptor to infect cells. This review summarizes the current knowledge available on cellular CHIKV receptors and the attachment factors used by CHIKV.

## 1. Chikungunya Virus (CHIKV)

**Figure 1:**
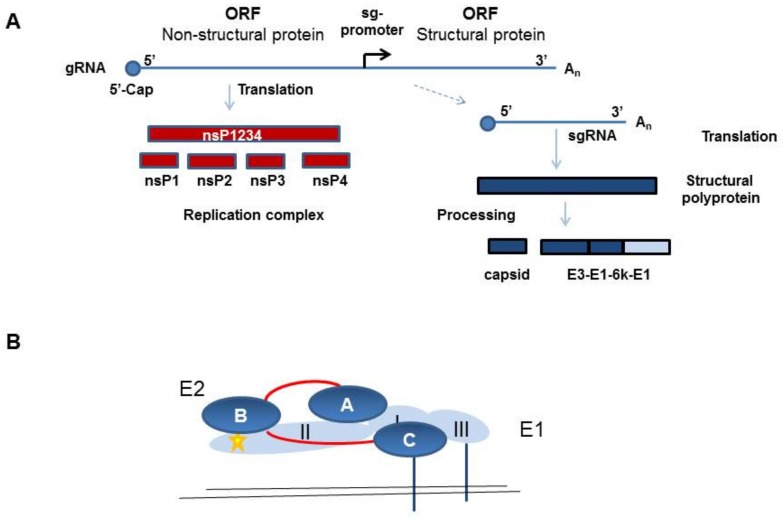
Schematic illustration of the chikungunya virus (CHIKV) protein expression and the envelope protein E2/E1 protein structure. (**a**) Schematic overview of the CHIKV non-structural and structural protein expression. Non-structural proteins (nsPs) are translated from the gRNA and structural proteins from the sgRNA, which is synthesized from the minus-strand RNA, which is not depicted here. (**b**) Illustration of the E2 (dark blue) and E1 (light blue) structure as a heterodimer. The β-ribbon connector is depicted in red and the fusion peptide in yellow.

CHIKV belongs to the family of Togaviridae, genus *Alphavirus*. In humans, infection with CHIKV causes chikungunya fever. In the acute phase, this is a febrile illness with high fever, rash, and headache, and it is associated with a high rate of acute and chronic long-lasting polyarthralgia. Alphaviruses have a single-strand positive polarity RNA genome, which encodes two polyproteins, namely, the non-structural proteins 1–4 (nsP1–4) and the structural proteins. The structural protein precursor is proteolytically cleaved into capsid (C), the envelope proteins E1, E2, and E3, and the small 6K protein (Figure 1A).

Heterodimers of the E1 and E2 proteins assemble into spikes on the virion surface and facilitate the infection of target cells [1]. The E1 protein contains a hydrophobic fusion peptide and is necessary for viral and cellular membrane fusion. The E2 protein is thought to be responsible for receptor binding, because it is the main target of neutralizing antibodies. E2 is synthesized as the precursor p62, which still contains covalently bound E3. The p62 protein is subsequently cleaved in the trans-Golgi apparatus by furin to remove E3. However, E3 can remain electrostatically bound to E2 until it is finally released during virus maturation, whereas E2 stays on the surface and is anchored in the viral membrane. E2 and E1 form heterodimers with E2 covering the fusion peptide in E1 to prevent premature fusion, and these dimers form trimers, which are the spikes on the virus surface [1] (Figure 1B). The E1 protein is a type II membrane protein and contains three β-barrel domains. Domain I is between domains II and III, and the fusion loop is at the distal end of domain II [1] (Figure 1B). The E2 protein belongs to the immunoglobulin superfamily and has three immunoglobulin domains—domain A is in the center, domain B is at the end of the spike, and domain C is membrane proximal and hidden from the virus surface. Domain B is at the tip of a long β-ribbon connector which connects it with domain A and C (Figure 1B). Structural analyses have indicated that in mature alphavirus particles, the E2 protein domain A makes three-fold contacts at the top of the spike and domain B faces to the side, giving the spike a propeller-like shape [1]. 

Epitope mapping of antibodies induced by a CHIKV infection in humans has shown that the E2 protein is the main target of CHIKV-neutralizing antibodies [2,3,4,5]. Domain B and the adjacent acid-sensitive regions (ASRs), which are part of the β-ribbon connector, are also recognized by monoclonal antibodies that protect against CHIKV infection [6,7,8]. This region is rearranged at low pH to expose the fusion loop in the E1 protein that is required for CHIKV entry into cells, and IgG binding may interfere with these rearrangements [9].

## 2. Virus Cell Entry

Infection of a target cell starts with the attachment of the virus to the cell surface. Here, two types of consequences can occur, namely, attachment and entry promotion. The binding of the virus to cells concentrates viral particles on the cell surface; however, attachment factors do not necessarily trigger the conformational changes in the envelope protein that allow virus cell entry. Therefore, attachment factors are usually non-specific and can be used by diverse types of viruses. In contrast, canonical virus receptors promote virus entry and, characteristically, their binding induces a conformational change in the viral envelope glycoproteins that is needed for membrane fusion and the release of the capsid and genetic material into the cytoplasm. Additionally, receptor-mediated routing of the virus into low pH vesicles can promote membrane fusion [10]. Virus–receptor interactions are therefore very specific and determine the host range of the virus. As alphaviruses are transmitted by arthropod hosts, it is expected that the viruses use either a highly evolutionarily conserved receptor or different entry mechanisms for insect and mammalian cells.

**Figure 2:**
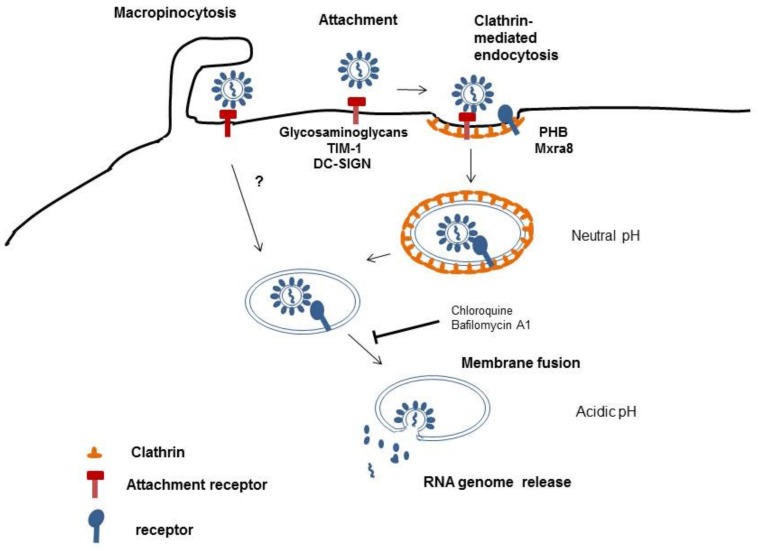
Schematic illustration of CHIKV cell entry.

Details of how alphaviruses enter host cells are still not completely resolved; however, alphaviruses have been reported to be taken up by clathrin-mediated endocytosis [10,11,12] (Figure 2). Endocytic vesicles coated with clathrin are able to rapidly traverse the cell membrane and deliver cargo into the cytoplasm. The acidic pH in endosomes triggers penetration and uncoating of alphaviruses [10]. However, this may not be the only pathway for alphaviruses to infect cells. Other authors have shown that CHIKV can enter cells via a clathrin-independent, epidermal growth factor receptor substrate 15 (Eps15)-dependent pathway. They demonstrated this using knockdown of Eps15 and clathrin heavy chain, a major scaffold protein of the clathrin coat [13]. This suggests that several pathways are used by CHIKV to facilitate its entry into target cells. After entering the endosomal compartment, fusion of CHIKV with the host cell membrane depends on a low pH environment as lysomotropic agents, like chloroquine or bafilomycin A1, considerably inhibit CHIKV infection [13], [14]. Recently, macropinocytosis was reported to be an entry pathway for CHIKV into human muscle cells [15]. Macropinosomes are large, uncoated vesicles involved in unspecific uptake of extracellular material. Their formation is actin-dependent and is initiated by the stimulation of growth factor receptors by the virus. This results in signal transduction in the host cells and actin filament polarization, which pushes the membrane forward to form ruffles. Some of these ruffles fold inwards and fuse with the cell membrane forming macropinosomes that take up bound viruses [16] (Figure 2).

## 3. Cellular Proteins Interacting with CHIKV

Several attachment factors have been described for CHIKV and other alphaviruses. Among the central factors are glycosaminoglycans (GAGs). GAGs are large complex carbohydrate molecules that are an essential part of the extracellular matrix and are ubiquitously expressed at the cell surface of most mammalian cell types. GAGs include, among others, heparan sulfate, keratan sulfate, chondroitin sulfate, and dermatan sulfate [17]. The role of GAGs in alphavirus attachment has been discussed extensively by others [18,19,20,21,22,23]. Point mutations within the E2 protein (e.g., E79K, G82R, or E166K) have been found in attenuated vaccine strains that were cell culture-adapted and showed enhanced GAG dependency but reduced in vivo replication [24,25,26]. These mutations mainly increase the positive charge in domain A of the E2 protein and affect the binding affinity of the virus [25]. However, cell-surface GAGs are not absolutely necessary for CHIKV infection, but rather promote viral entry and thereby replication. There are also GAG-independent entry pathways, as CHIKV entry into GAG-deficient cells is still possible, and soluble GAGs cannot fully block CHIKV cell entry [23]. As expected for an attachment factor, GAGs mainly enhance alphavirus infection.

The cell-surface glycoprotein T-cell immunoglobulin and mucin 1 (TIM-1) is expressed on a large variety of cells. It has been demonstrated to be a virus receptor for a multitude of viruses, including filoviruses, and correspondingly has also been found to support alphavirus cell entry [27,28]. TIM-1 binds phosphatidylserine (PtdSer) located in the viral membrane and this binding may also mediate virus internalization. CHIKV entry has been shown to be moderately enhanced in TIM-1-overexpressing cells, and transduction of these cells by CHIKV-pseudotyped vectors can be partially inhibited by PtdSer liposomes [28]. The deletion of 90 amino acids of the TIM-1 stalk region showed that a functional PtdSer-binding domain appropriately spaced from the plasma membrane is sufficient to enhance virus entry [28]. In addition, other PtdSer-binding proteins, such as Axl and TIM-4, can promote CHIKV infection in a similar way [29]. Since the cytoplasmic and transmembrane domains of TIM-1 are not essential for enhancing virus entry, the concept has evolved that the TIM receptor family are attachment factors that enhance infections rather than being specific receptors [30]. Likewise, the C-type calcium-dependent lectin DC-SIGN (DC-specific intercellular adhesion molecule-3-grabbing non-integrin) acts as an attachment factor for several viruses and its expression has been shown to significantly enhance the infection of cells by the alphaviruses Semliki Forest virus (SFV) and CHIKV [31]. Furthermore, polymorphisms in the DC-SIGN gene appear to influence the risk of developing clinical symptoms for CHIKV-infected patients [32].

Other cellular proteins interacting with the CHIKV E2 or E1 proteins have been identified by high-throughput yeast two-hybrid screens using a human fetal brain cDNA library. This screen is based on cytoplasmic protein–protein interactions with the limitation that not all post-translational modifications are present in the proteins like glycosylation and proper disulfide bounds. Actin gamma 1, collagen type I-alpha-2, and tyrosine phosphatase, non-receptor type 2 (PTPN2) have been identified as E2-binding partners; however, functional studies are still lacking [33]. Some mechanistic data are available from studies with another alphavirus, Ross River virus (RRV). RRV has been reported to use the collagen-binding α1β1 integrin as a cellular receptor. RRV infection could be inhibited by collagen IV and antibodies specific for the β1 and α1 integrin proteins, and fibroblasts from α1-integrin knockout mice were less efficiently infected than fibroblasts from wild-type mice [34].

Several genome-wide loss-of-function screens have been performed for CHIKV and other alphaviruses, and factors involved in virus entry have been found. In a small interfering RNA (siRNA) screen of human osteosarcoma cells, two proteins involved in the endocytosis pathway were identified as entry factors for CHIKV. Although a complete shut-off of gene expression by siRNA is difficult, fuzzy homologue (FUZ) has been identified. FUZ is involved in cell polarity and cilia biogenesis, and is required for the clathrin-dependent internalization of alphaviruses. The second identified protein, tetraspanin membrane protein 9 (TSPAN9), modulates the early endosome compartment, making it more permissive for membrane fusion of viruses penetrating via early endosomes like CHIKV and SFV [35]. Although direct binding of CHIKV particles to TSPAN9 has not been demonstrated [35], TSPAN9 depletion has been shown to strongly inhibit infection not only of alphaviruses but also of vesicular stomatitis virus (VSV) [36]. VSV and alphaviruses fuse in early endosomes; therefore, it is likely that TSPAN9 acts by modulating the early endosomes to make them more permissive for membrane fusion [36].

This loss-of-function screening has also identified factors that negatively affect CHIKV infection [35]. Interferon-induced transmembrane protein 3 (IFITM3) is a membrane-localized cellular restriction factor that blocks fusion between virus and host membranes and inhibits virus entry [37]. It has recently been reported that IFITM3-knockout mice show more severe pathologies than wild-type mice after CHIKV infection [38]. Tetherin/BST-2 is another interferon-induced cellular membrane protein that negatively affects CHIKV infections. It inhibits the release of many enveloped viruses by directly tethering budded particles to the cell surface. Alphaviruses bud from cells and tetherin has been described to inhibit the release of SFV and CHIKV particles from host cells [39]. However, the CHIKV non-structural protein 1 (nsP1) has been shown to overcome tetherin-mediated tethering and downregulate tetherin expression [40]. In addition, increased viral load at the inoculation site, resulting in higher viremia and increased lymphoid tissues tropism, has been observed in tetherin-deficient mice in vivo, indicating that tetherin protects lymphoid tissues from CHIKV infection [41].

Another approach toward the identification of a potential CHIKV receptor made use of immobilized membrane proteins from permissive cell lines. These denatured proteins were incubated with CHIKV, and then the cellular proteins bound by the virus were identified by mass spectroscopy. This process led to the identification of prohibitin (PHB) proteins 1 and 2 [42]. Antibodies directed against PHB-1 or its downregulation by siRNA treatment slightly inhibited CHIKV infection, and binding of PHB-1 to the CHIKV E2 protein was detected by immunoprecipitation. PHB-1 is a ubiquitously expressed protein located at the cell membrane, the nucleus, and mitochondria [43]. PHBs are evolutionarily conserved and thereby fulfill the requirements of a CHIKV receptor. Since interference with PHB did not fully inhibit CHIKV infection, it is again likely that PHB-1 is only an infection-enhancement factor or that multiple pathways are available for CHIKV to enter cells.

All cellular molecules described so far were identified from mammalian cells. However, the life cycle of CHIKV includes a mosquito transmission step. Consequently, mosquito cells have also been screened for potential CHIKV receptors. Through a combination of virus overlay protein-binding assays and mass spectroscopy, ATP synthase β subunit (ATPSβ) was found to interact with CHIKV E2 protein in mosquito cells. These ATPases are mitochondrial and plasma membrane-bound protein complexes that associate ATP synthesis with the transport of protons across the membrane. Interference with ATPSβ by antibody inhibition or siRNA-mediated downregulation resulted in a partial reduction in viral entry and virus production, indicating that ATPSβ is involved in the CHIKV entry process in mosquitos [44]. The ATPSβ gene is highly conserved; however, its involvement in CHIKV entry in mammalian cells has not yet been studied.

Recently, a genome-wide CRISPR-Cas9-based screen identified the cell adhesion molecule Mxra8 as an entry mediator for multiple arthritogenic alphaviruses, including CHIKV, RRV, and Mayaro virus (MAYV) and O’nyong-nyong virus (ONNV). The CRISPR-Cas9 system results in a complete knockout of the gene of interest and its protein synthesis and is superior to the siRNA-based gene knockdown used in the screens described above. CHIKV particles bound directly to Mxra8, and this led to enhanced virus attachment and internalization into cells [45]. A fusion protein of the extracellular domain of Mxra8 and IgG-Fc fragment (Mxra8-Fc) or anti-Mxra8 monoclonal antibodies were able to block CHIKV infection [45]. However, residual CHIKV infection was also detectable in vitro and in vivo in the absence of Mxra8 [45]. There is no mosquito orthologue of Mxra8, which suggests that additional unidentified host factors exist that support cell binding and entry into mosquito cells. In addition, Mxra8-deficient mice showed decreased infection of musculoskeletal tissues with CHIKV, MAYV, RRV, or ONNV [46]. Moreover, a recombinant CHIKV with reduced binding to Mxra8 was attenuated in vivo in wild-type mice and CHIKV infection was enhanced in transgenic flies expressing Mxra8 [47]. These studies demonstrate a role for Mxra8 in the pathogenesis of alphaviruses; however, they show that Mxra8 is not an exclusive alphavirus receptor. Although reduced alphavirus infection was observed, alphavirus replication clearly occurred in the absence of Mxra8 [46].

Two different binding modes of Mxra8 to viral particles have been determined by structural analyses. Only high-affinity binding sites are bound by Mxra8 when E3 is still attached to E2. When E3 has been released from the CHIKV virion, occupancy of both high- and low-affinity binding sites can be detected [46]. These observations revealed for the first time that E3 affects receptor binding [47,48]. For some alphaviruses the E3 protein remains covalently or non-covalently associated with the mature virus. In Sindbis virus and Venezuelan equine encephalitis virus (VEEV), E3 is attached to virions and both viruses are only mildly dependent on Mxra8 for cell entry. CHIKV strain-specific differences in Mxra8 dependency have also been observed that may be due to preferred virus entry via GAG binding or the presence of a more mature virus after E3 has been released and is absent [45,47,48].

**Figure 3:**
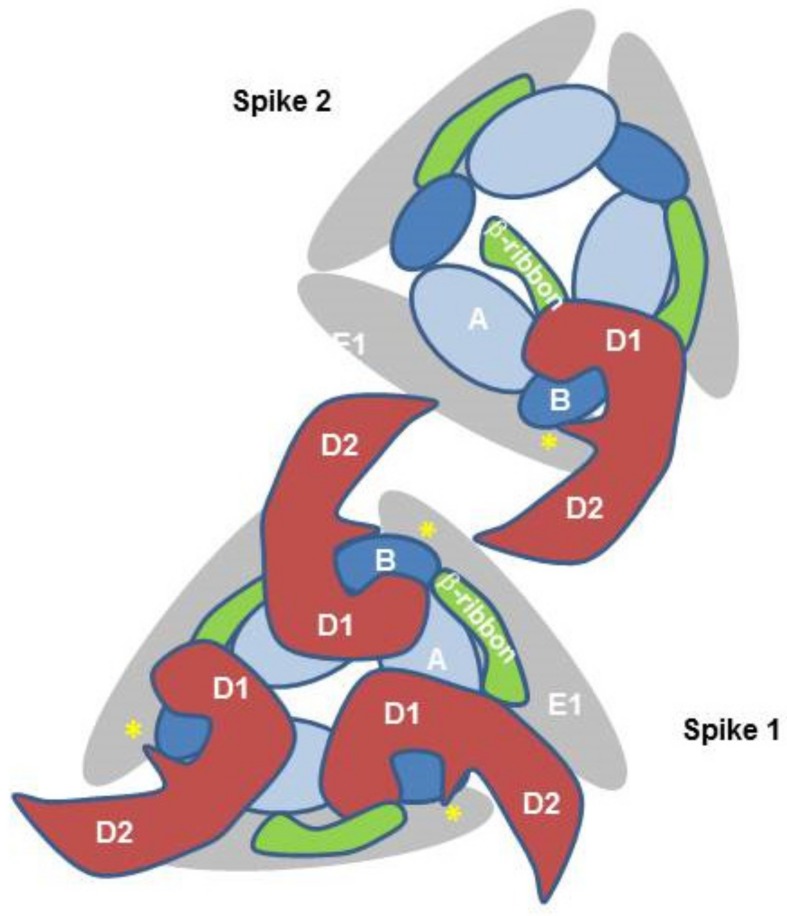
Schematic illustration of CHIKV–E2–E1–Mxra8 interaction. The simplified schematic representation is showing two CHIKV E2/E1 trimeric spikes viewed from the top. Mxra8 binding crosslinks CHIKV spikes and Mxra8 interacts with E2 and E1. E2 domain B is depicted in dark blue, E2 domain A in light blue, and the β-ribbon connector in green. The Mxra8 molecule with domains D1 and D2 is depicted in red. In addition, the E1 Domain II is in grey and the fusion loop is in yellow. For simplicity, only one Mxra8 molecule is shown in spike 2.

The crystal structure of Mxra8 in complex with the CHIKV E2/1 proteins and the cryo-electron microscopy structure of human Mxra8 and a CHIKV virus-like particle have revealed novel structural details of the binding. Mxra8 has two Ig-like domains, namely, D1 and D2. They are oriented head-to-head and D2 is inserted between two discontinuous fragments of D1 and two hinge loops are formed. Mxra8 binds in a groove created by two neighboring CHIKV E2-E1 heterodimers from one trimeric spike on the surface of the virion [47,48]. Three Mxra8 proteins bind to one trimeric spike (Figure 3). Mxra8 interacts with two adjacent E2/E1 dimers with distinct binding sites, involving E2 domains A and B and the ß-ribbon connector and E1 (Figure 3). In contrast to previous assumptions concerning receptor molecules, Mxra8 interacts with both the E2 and the E1 protein. This binding crosslinks CHIKV spikes in a similar manner to a broadly neutralizing antibody [6]; however, in the context of Mxra8, this binding facilitates attachment and entry of the virus, although Mxra8 binding does not induce a substantial conformational change in E2 [47,48]. Mxra8 apparently directs the virus to the low pH environment of the endosome and this additionally triggers the release of the capsid into the cytoplasm.

## 4. Conclusions

As summarized here, several factors enhancing CHIKV infection have been characterized, although none of them is a unique CHIKV receptor. CHIKV infection of factor-deficient cells is still possible, although at very low rates. This presents the argument for multiple entry pathways, some of which are highly efficient, such as that involving Mxra8, whereas others are less efficient but can be used by the virus if the primary receptor is not available. Therefore, the hunt for entry factors continues—there is still much to do.
